# Coenzyme Q_10_, *α*-Tocopherol, and Oxidative Stress Could Be Important Metabolic Biomarkers of Male Infertility

**DOI:** 10.1155/2015/827941

**Published:** 2015-02-25

**Authors:** Anna Gvozdjáková, Jarmila Kucharská, Jozef Dubravicky, Viliam Mojto, Ram B. Singh

**Affiliations:** ^1^Pharmacobiochemical Laboratory, Third Medical Department, Medical Faculty, Comenius University in Bratislava, Sasinkova Street 4, 811 08 Bratislava, Slovakia; ^2^Urology Department, Comenius University in Bratislava, Limbová Street 5, 833 05 Bratislava, Slovakia; ^3^Third Medical Department, Medical Faculty, Comenius University in Bratislava, Limbová Street 5, 833 05 Bratislava, Slovakia; ^4^Halberg Hospital and Research Institute, Moradabad 244001, India

## Abstract

Oxidative stress, decreased antioxidant capacity, and impaired sperm mitochondrial function are the main factors contributing to male infertility. The goal of the present study was to assess the effect of the per os treatment with Carni-Q-Nol (440 mg L-carnitine fumarate + 30 mg ubiquinol + 75 IU vitamin E + 12 mg vitamin C in each softsule) in infertile men on sperm parameters, concentration of antioxidants (coenzyme Q_10_,  CoQ_10-TOTAL_, *γ*, and *α*-tocopherols), and oxidative stress in blood plasma and seminal fluid. Forty infertile men were supplemented daily with two or three Carni-Q-Nol softsules. After 3 and 6 months of treatment, improved sperm density was observed (by 48.9% and 80.9%, resp.) and after 3-month treatment the sperm pathology decreased by 25.8%. Concentrations of CoQ_10-TOTAL_ (ubiquinone + ubiquinol) and *α*-tocopherol were significantly increased and the oxidative stress was decreased. In conclusion, the effect of supplementary therapy with Carni-Q-Nol showed benefits on sperm function in men, resulting in 45% pregnancies of their women. We assume that assessment of oxidative stress, CoQ_10-TOTAL_, and *α*-tocopherol in blood plasma and seminal fluid could be important metabolic biomarkers in both diagnosis and treatment of male infertility.

## 1. Introduction

Infertility is a disease of the reproductive system defined by the failure to achieve a clinical pregnancy after twelve months or more of regular unprotected sexual intercourse. Infertility of men is one of the major stressful factors, implicated in 30–40% of couples. Imbalance between increased reactive oxygen species (ROS) production and decreased antioxidant capacity (oxidative stress) in the male reproductive tract contribute to the infertility and can cause a defect in sperm function. Oxidative stress in the testes or semen can be attributed to infection, inflammation, smoking, industrial exposure, chemotherapy, drugs, and varicocele, as well as leukocytes, which are primary producers of endogenous oxidants in semen [1–4].

In human reproduction, only a minimum amount of ROS is needed to regulate sperm physiological processes such as capacitation, acrosome reaction, and the signaling process to ensure fertilization [5]. In physiological conditions, the seminal plasma contains antioxidant mechanisms that protect spermatozoa against oxidative stress. Excessive ROS production has been correlated with a reduction of sperm motility and amount, increased pathology of sperm morphology, and decreased sperm mitochondrial adenosine triphosphate (ATP) production, resulting in male infertility. High levels of ROS were detected in semen samples in 25% to 40% of infertile men [6]. Our previous study reported increased plasma levels of malondialdehyde and decreased sperm mitochondrial ATP production in patients with oligozoospermia [7]. For sperm mitochondrial energy production sufficient coenzyme Q_10_ (CoQ_10_) and carnitine concentrations are important.

Three redox states of CoQ_10_ in Q-cycle occur in the organism: ubiquinone (CoQ_10_-oxidized), ubiquinol (CoQ_10_H_2_-reduced), and semiquinone (partially reduced, as radical). Ubiquinol is a strong lipophilic antioxidant; it can regenerate other antioxidants such as vitamin E and vitamin C. The ubiquinol concentration in the body is approximately 90% of the total CoQ_10_. A strong correlation has been reported to exist between sperm count, motility, and ubiquinol concentration in seminal fluid [8]. CoQ_10_ biosynthesis is very active in the testes and high levels of ubiquinol are present in sperm [9, 10]. Our previous study found a direct correlation between seminal plasma CoQ_10-TOTAL_ concentration and sperm motility [11].

Other important sperm components naturally occurring in the body are carnitines. Carnitines provide energy for spermatozoa and affect sperm motility and maturation. They also function as antioxidants providing protection against ROS. Carnitine supplementation has been demonstrated to improve sperm concentration, mobility, viability, morphology, and total antioxidative capacity [12]. In recent years, intensive research has been focused on various antioxidants for treatment of human fertility disturbances, considering also their optimal doses and combinations.

The effect of supplementary therapy with ubiquinol-carnitine combination in male infertility is not known. The aim of our study was to evaluate the effect of Carni-Q-Nol (carnitine, ubiquinol, vitamin E, and vitamin C) treatment of infertile men on sperm parameters, oxidative stress, concentration of antioxidants (CoQ_10-TOTAL_, *α*-tocopherol, and *γ*-tocopherol), and rate of pregnancies in the women of treated infertile men.

## 2. Materials and Methods

### 2.1. Patients

In this study, 40 infertile men with oligoasthenozoospermia, aged 28–40 years, signed an informed consent prior to their enrolment. Patients with azoospermia were excluded. The subjects had not taken CoQ_10_ or carnitine before the study for at least four weeks. Infertile men were per os supplemented Carni-Q-Nol, produced by Tishcon Corp., Westbury, New York, USA. Each softsule of Carni-Q-Nol contained 440 mg L-carnitine fumarate + 30 mg ubiquinol + 75 IU vitamin E + 12 mg vitamin C. During the first 3 months, the daily dose of Carni-Q-Nol was 2 softsules; during the next 3 months, 3 softsules were taken daily. A limitation of the study was the lack of a control group for ethical reasons. Before study, women of subfertile men had lower values of plasma alpha-tocopherol and higher lipid peroxidation in comparison with control women.

### 2.2. Seminal Fluid Analysis

Seminal fluid was collected after 3–5 days of sexual abstinence. Seminal fluid analysis, as well as sperm concentration, motility, and morphology in male subjects, was assessed in accordance with WHO criteria used for diagnosis of male infertility [13]. The normal values include >20 × 10^6^/mL concentration with grade *a* motility in 25% or grade *a* + *b* motility in 50% of spermatozoa and normal morphology in at least 30% of the spermatozoa. Motility was graded as follows: classes *a* and *b*: fast and weak forward motility; class *c*: nonprogressive motility [13]. Data of seminal fluid analysis were received from two seminal fluid samples before as well as after treatment.

### 2.3. Biochemical Analysis

Blood plasma and seminal fluid concentrations of CoQ_10-TOTAL_, *α*-tocopherol, and *γ*-tocopherol were determined by HPLC method using a UV detector at 275 and 295 nm and calculated by external standards (Sigma) [14]. Minor modifications were made in seminal fluid preparation as follows: 1.0 mL seminal fluid + 200 *μ*L* p*-benzoquinone (18.5 mmol·L^−1^) + 2.0 mL methanol + 1.0 mL (100 mmol·L^−1^) sodium dodecyl sulphate, vortexed 1 min, with 3.0 mL and repeatedly with 2.0 mL hexane, vortexed 5 min, and centrifuged for 10 min [15, 16]. The organic phases were collected and evaporated under gas nitrogen; the residue dissolved in 50 *μ*L of ethanol, and 20 *μ*L was injected on the column SGX C18 7 *μ*m (Tessek). Elution was performed with methanol/acetonitrile/ethanol (6/2/2; v/v/v; Merck), flow rate 0.90 mL·min^−1^. Thiobarbituric acid reactive substances (TBARS)—oxidative stress parameter—were determined spectrophotometrically [17].

### 2.4. Statistical Analysis

The data are expressed as mean ± standard error of the mean. Statistical significance of differences between baseline values and 3- and 6-month treatment was evaluated using one-way analysis of variance (ANOVA), multiple comparisons with Bonferroni correction, and paired Student's *t*-test. Statistical significance was considered at *P* < 0.05.

## 3. Results

### 3.1. Comparison of Sperm Parameters, Antioxidants, and TBARS in Infertile Men with Different Sperm Motility

Significant differences were found in* sperm density* between groups of sperm motility grades *a* + *b* and *b* + *c* (*P* < 0.001) and in* sperm pathology a* + *b* versus *b* + *c* (*P* < 0.001).* In seminal fluid,* concentrations of CoQ_10-TOTAL_ and *α*-tocopherol were significantly higher in groups *a* + *b* versus *b* + *c*by 36.1% (*P* < 0.01) and by 33.0% (*P* < 0.01), respectively. Concentrations of TBARS in seminal fluid were similar in both groups.* In blood plasma* of infertile men, concentrations of CoQ_10-TOTAL_ and *α*-tocopherol were significantly lower in groups *a* + *b* versus *b* + *c* (−39.6%); *P* < 0.001 and (−33.9%) *P* < 0.001, respectively. In both groups concentrations of TBARS in plasma were without significant differences, yet, in both groups of infertile men, TBARS were increased by 21.5% and 19.0% in comparison with reference values, ≤4.50 *μ*mol·L^−1^ (Table 1).

### 3.2. Effect of Carni-Q-Nol on Sperm Parameters, Antioxidants, and TBARS of Infertile Men

After 3 months of treatment with Carni-Q-Nol the sperm density increased by 39.8% (*P* < 0.001) and after 6 months by 78.0% (*P* < 0.001). Sperm pathology significantly decreased after 3 months by 25.8% (*P* < 0.001). Unfortunately, we have only incomplete data of the sperm pathology after 6 months of treatment, which could thus not be included in the evaluation. Sperm analysis did not continue in cases of confirmed pregnancies of female partners. After Carni-Q-Nol treatment, there were no significant changes in CoQ_10-TOTAL_ in seminal fluid. Concentrations of *α*-tocopherol significantly decreased after 3 and 6 months of supplementary therapy (*P* < 0.05), and *γ*-tocopherol significantly decreased in seminal fluid after 6 months of therapy (*P* < 0.05). TBARS in seminal fluid decreased significantly in comparison with baseline values by 12.3% (*P* < 0.05) after 6-month therapy. In blood plasma, the concentration of CoQ_10-TOTAL_ significantly increased after 3 or 6 months of supplementary therapy by 131.0% (*P* < 0.001) and by 108.9% (*P* < 0.05), respectively, in comparison with baseline values. The *α*-tocopherol concentration significantly increased by 27.5% (*P* < 0.001) after 3-month and by 20.3% (*P* < 0.05) after 6-month supplementation. The levels of *γ*-tocopherol decreased both after 3 and 6 months of supplementary therapy (−50.7%, *P* < 0.001, or −46.8%, *P* < 0.01). Baseline values of TBARS were higher than the reference values and decreased both after 3-month (*P* < 0.05) and after 6-month supplementation (*P* < 0.01, −13.3%) in comparison with baseline values (Table 2).

### 3.3. Safety Assessment

Carni-Q-Nol oral administration was well tolerated, without any side effects.

### 3.4. Pregnancies of Women

Pregnancies of women were confirmed in 45% after Carni-Q-Nol treatment of infertile men. After one month of treatment 2 pregnancies, after 3 months of treatment 4 pregnancies, and between 5 and 6 months of treatment 12 pregnancies occurred. In three of these pregnancies the in vitro fertilization (IVF) method was used. Before initiation of this study, 6 women were on the IVF 1–3 times without any success; 2 of them were on thyroid gland therapy. A part of these results was presented [18, 19]. We cannot compare pregnancy rate in our study to normal pregnancy rate of healthy couples; we do not have such data.

## 4. Discussion

Infertility affects 10–15% of couples and nearly 80 million couples worldwide. One of the major causes of defective sperm function and sperm motility is oxidative stress. A review provided a summary of evidence for the presence of oxidative stress in human spermatozoa [20]. The principal sources of endogenous ROS in semen are leukocytes and abnormal spermatozoa. Recently a hypothesis has been suggested that the damage of sperm DNA is caused mainly by mitochondrial ROS originating from damaged spermatozoa [21]. In male infertility, a decreased sperm mitochondrial ATP production [22] and increased mitochondrial DNA fragmentation were found in correlation with decreased sperm motility [21]. Abnormal and nonviable spermatozoa can generate additional ROS and reactive nitrogen species, which can disrupt normal sperm development and may result in apoptosis [23]. Mitochondrial ROS coming from defective spermatozoa attack sperm DNA. High sperm DNA fragmentation could be associated with higher rates of pregnancy loss after IVF, IVF-ICSI (intracytoplasmic sperm injection), and ART (assisted reproductive technology) treatment. There is strong evidence that supplementation with antioxidants improves sperm motility [20, 23].

In our study baseline blood plasma TBARS levels of infertile men were increased in comparison with reference values (Tables 1 and 2). We detected significant differences in sperm density and sperm pathology between sperm motility groups *a* + *b* and *b* + *c*, corresponding with increased plasma TBARS in infertile men. In group *b* + *c* with reduced sperm motility, decreased CoQ_10-TOTAL_ and *α*-tocopherol concentrations in seminal fluid of infertile men were found as compared with *a* + *b* group (Table 1) [11].

Over the last years, intensive research has been focused on various antioxidants and their optimal doses and combinations, for more effective and safe treatment of human fertility disturbances [20]. Improvement of sperm parameters after antioxidant therapy of infertile men (with vitamin E, vitamin C, N-acetyl-L-cysteine, carnitine, or CoQ_10_) may result in higher pregnancy rates [2, 24]. Vitamin E as an antioxidant may directly quench free radicals and together with CoQ_10_ protect lipid membranes from peroxidative damage [24]. Vitamin E is able to reduce seminal ROS levels in men with infertility. Combined therapy with vitamin C resulted in improvement of DNA fragmentation yet excessive intake of nutraceuticals can have also negative consequences [2].

A number of clinical studies documented the beneficial effect of CoQ_10-OX_-ubiquinone treatment in male infertility, with various daily doses and durations of treatment. Coenzyme Q_10_ treatment improved semen quality and motility in men with idiopathic infertility, and its concentration both in seminal plasma and in sperm cells was increased. Moreover, a direct correlation between CoQ_10_ concentrations and sperm motility was found [25]. When men with idiopathic oligoasthenoteratozoospermia infertility were supplemented with ubiquinone in a daily dose of 200 mg during 26 weeks, an increase in sperm density by 30.7%, sperm motility by 24.3%, and sperm morphology by 33.3% was observed [26]. Recently, patients with idiopathic asthenozoospermia were supplemented with CoQ_10_ (200 mg/day) and D-Asp (2,660 mg/day) for three months. Significant improvement of sperm motility and protective effect of CoQ_10_ against oxidative stress and sperm DNA damage was proved [27]. In another study, men with idiopathic infertility were treated with 300 mg CoQ_10_ twice daily for 12 months. CoQ_10_ supplementation improved semen quality with beneficial effects on pregnancy rates [28]. Twenty-two men with idiopathic asthenozoospermia received 200 mg of CoQ_10_/day for 6 months. The authors observed significant improvement in sperm motility and forward motility [29]. Infertile men with varicocele were supplemented with 100 mg CoQ_10_ per day during 3 months. CoQ_10_ therapy improved semen parameters and antioxidant status in these patients [30]. Our previous study showed beneficial effect of ubiquinone in infertile men; a daily dose of 90 mg given for 3–9 months led to improvement of male fertility and increased pregnancy rates [31].

Ubiquinol (CoQ_10-RED_) is a stronger antioxidant in comparison with ubiquinone. A significant correlation between ubiquinol content and sperm count and motility in seminal fluid of infertile men was reported [8]. When infertile men were treated with 150 mg ubiquinol daily during four months, total sperm count was increased by 53% and sperm motility by 26% and sperm morphology was also improved [32]. Ubiquinol as supplementary therapy was used in men with idiopathic infertility, in a daily dose of 200 mg during 26 weeks. A positive effect of ubiquinol therapy was observed in sperm density improvement by 81.6%, sperm motility by 31.7%, and sperm morphology by 24.0% [33].

Our study showed a beneficial effect of treatment with a combination of antioxidants (carnitine with ubiquinol, vitamins E and C) on sperm density, which increased by 39.8% after 3 months of treatment and by 78.0% after 6 months of treatment and sperm motility was improved. Sperm pathology decreased in 25.8% after 3-month treatment. Carni-Q-Nol treatment significantly increased blood plasma CoQ_10-TOTAL_ concentration in comparison with baseline values: after 3 months of treatment an increase by 131.0% was observed and after 6 months of treatment there was an increase by 108.9%. In blood plasma, increased *α*-tocopherol concentrations yet decreased *γ*-tocopherol concentrations were found. The exact mechanism of *γ*-tocopherol decrease is not fully known, though it may be related to *α*-tocopherol administration. Carni-Q-Nol treatment significantly decreased TBARS in seminal fluid and blood plasma (Table 2). We suppose that the improvement of antioxidant capacity and the reduction of oxidative stress in infertile men after antioxidants treatment could be related to the improvement of sperm density, sperm motility, and reduction of sperm pathology.

**Figure 1:**
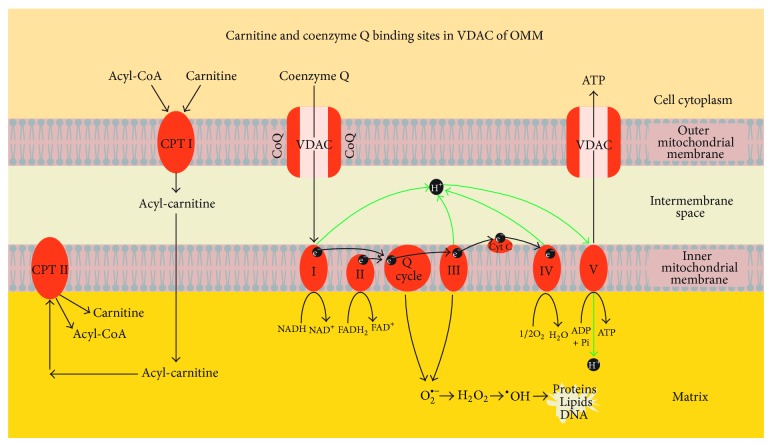
Proposed mechanism of coenzyme Q_10_ and carnitine effect in sperm mitochondria. Acyl-CoA: acyl-coenzyme A; CPT I: carnitine phosphate transpeptidase I; CPT II: carnitine phosphate transpeptidase II; CoQ: coenzyme Q; VDAC: voltage dependent anion channel; ATP: adenosine triphosphate; ADP: adenosine diphosphate; Pi: inorganic phosphate; I, II, III, IV, and V: respiratory chain complexes; H^+^ proton; e^−^: electron; Q-cycle: coenzyme Q cycle; cyt c: cytochrome c; NADH: reduced nicotinamide adenine dinucleotide; NAD^+^: nicotinamide adenine dinucleotide; FADH_2_: reduced flavin adenine dinucleotide; FAD: flavin adenine dinucleotide; O_2_^∙−^: superoxide radical; H_2_O_2_: hydrogen peroxide; OH^*∙*^: hydroxyl radical; H_2_O: water; O_2_: oxygen.

Mitochondrial CoQ functions include regulation of electron transport in the respiratory chain, receiving electrons from complex I and complex II and passing them to complex III, and transfer of protons from fatty acids to the matrix. An alternative function of CoQ may also be regulation of permeability transition pore opening and nutrition uptake through the voltage dependent anion channel (VDAC) of the outer mitochondrial membrane (OMM) [34]. The effect of L-carnitine and acetyl-L-carnitine alone or in combination significantly reduced oxidative stress. Carnitines provide energy for spermatozoa and affect sperm motility and maturation. They also function as antioxidants providing protection against ROS [12]. The mechanism of the beneficial effect of carnitine-ubiquinol combination on sperm mitochondria of infertile men could occur through their uptake by VDAC of the outer mitochondrial membrane (OMM), along with the activity of carnitine palmitoyl transferase I (CPT I) in OMM (Figure 1). Further studies are required to support this hypothesis.

## 5. Conclusions

In conclusion, our results documented the importance of assessment indicators of oxidative stress, antioxidant status, and lipid peroxidation in plasma and seminal fluid for the diagnosis of male infertility. The effect of supplementary therapy with Carni-Q-Nol showed benefits on sperm function in men, resulting in 45% pregnancies of their women. We assume that assessment of oxidative stress, CoQ_10_, and *α*-tocopherol in blood plasma and seminal fluid could be important metabolic biomarkers in both diagnosis and treatment of male infertility.

## Figures and Tables

**Table 1:** Comparison of sperm parameters, antioxidants, and TBARS in infertile men with different sperm motility groups.

	Sperm motility	
	*a* + *b*	*b* + *c*
Sperm density (10^6^·mL^−1^)	24.58 ± 2.17	13.53 ± 1.76^***^
Sperm pathology (%)	33.75 ± 1.25	51.11 ± 2.54^***^

	Seminal fluid	
	*a* + *b*	*b* + *c*

CoQ_10-TOTAL_ (*μ*g·mL^−1^)	0.147 ± 0.012	0.094 ± 0.010^**^
*α*-Tocopherol (*μ*g·mL^−1^)	0.669 ± 0.086	0.448 ± 0.038^*^
TBARS (nmol·mL^−1^)	9.188 ± 0.460	8.979 ± 0.392

	Blood plasma	
	*a* + *b*	*b* + *c*

CoQ_10-TOTAL_ (*μ*mol·L^−1^)	0.434 ± 0.025	0.606 ± 0.037^***^
*α*-Tocopherol (*μ*mol·L^−1^)	24.44 ± 1.26	32.73 ± 1.58^***^
TBARS (*μ*mol·L^−1^)	5.467 ± 0.257	5.354 ± 0.171

Statistical significance: *b* + *c* versus *a* + *b*; ^*^ *P* < 0.05, ^**^ *P* < 0.01, and ^***^ *P* < 0.001. Reference values of antioxidants in plasma: CoQ_10-TOTAL_ 0.4–1.0 *μ*mol·L^−1^, *α*-tocopherol 15–40 *μ*mol·L^−1^, and TBARS < 4.5 *μ*mol·L^−1^.

**Table 2:** Effect of Carni-Q-Nol on sperm parameters, antioxidants, and TBARS of infertile men.

	Baseline	Carni-Q-Nol	
		3 months	6 months
Sperm density (10^6^·mL^−1^)	17.27 ± 1.88	24.15 ± 2.56^***^	31.94 ± 4.12^***^
Sperm pathology (%)	47.78 ± 3.64	35.46 ± 3.48^***^	No data

Seminal fluid			
CoQ_10-TOTAL_ (*μ*g·mL^−1^)	0.112 ± 0.011	0.096 ± 0.009	0.103 ± 0.013
*α*-Tocopherol (*μ*g·mL^−1^)	0.604 ± 0.081	0.434 ± 0.055^*^	0.383 ± 0.047^*^
*γ*-Tocopherol (*μ*g·mL^−1^)	0.039 ± 0.005	0.037 ± 0.005	0.030 ± 0.005^*^
TBARS (nmol·mL^−1^)	8.980 ± 0.342	8.950 ± 0.337	7.878 ± 0.588^*^
Blood plasma			
CoQ_10-TOTAL_ (*μ*mol·L^−1^)	0.552 ± 0.032	1.275 ± 0.092^***^	1.153 ± 0.179^*^
*α*-Tocopherol (*μ*mol·L^−1^)	27.76 ± 1.38	35.40 ± 1.39^***^	33.41 ± 3.59^*^
*γ*-Tocopherol (*μ*mol·L^−1^)	2.188 ± 0.156	1.079 ± 0.088^***^	1.163 ± 0.173^**^
TBARS (*μ*mol·L^−1^)	5.217 ± 0.157	4.774 ± 0.089^*^	4.525 ± 0.139^**^

Statistical significance: 3 months versus baseline; 6 months versus baseline; ^*^ *P* < 0.05, ^**^ *P* < 0.01, and ^***^ *P* < 0.001.

Reference values of antioxidants in plasma: CoQ_10-TOTAL_ 0.4–1.0 *μ*mol·L^−1^, *α*-tocopherol 15–40 *μ*mol·L^−1^, *γ*-tocopherol 2–7 *μ*mol·L^−1^, and TBARS < 4.5 *μ*mol·L^−1^.
